# Cortical activation characteristics during different swallowing tasks in post-stroke patients: a functional near-infrared spectroscopy study

**DOI:** 10.3389/fneur.2025.1733949

**Published:** 2026-01-15

**Authors:** Meng Guo, You Tang, Wenjing Liu, Yuxuan Zhang, Zihao Sun, Chunxiao Wan

**Affiliations:** 1 Department of Physical and Rehabilitation Medicine, Tianjin Medical University General Hospital, Tianjin, China; 2 Department of Rehabilitation, Binzhou Medical University Hospital, Binzhou, China; 3 Department of Joint Surgery, Binzhou People’s Hospital, Binzhou, China; 4 School of Special Education and Rehabilitation, Binzhou Medical University, Yantai, China; 5 College of Rehabilitation Medicine, Tianjin Medical University, Tianjin, China; 6 The Neuro-Regulation and Remodeling Laboratory, Tianjin Medical University General Hospital, Tianjin, China

**Keywords:** dysphagia, functional near-infrared spectroscopy, rehabilitation, stroke, swallowing

## Abstract

**Objective:**

This study employed functional near-infrared spectroscopy (fNIRS) to investigate cortical activation patterns in post-stroke dysphagia (PSD) patients compared with non-dysphagic patients during specific swallowing tasks.

**Methods:**

Twenty-nine patients with supratentorial stroke were recruited and divided into a dysphagic group (*n* = 14) and a control group (*n* = 15). Brain activity was monitored using fNIRS during single swallowing task (SST), continuous swallowing task (CST), and video-continuous swallowing dual-task test (DTT). Activation patterns were analyzed within and between groups using the general linear model (GLM) and block-average hemodynamic analysis.

**Results:**

In the dysphagia group, during the SST, activation was observed in the R-FPA, bilateral PSMC, and L-DLPFC (*p* < 0.05). During the CST, activation was observed in the R-PSMC (*p* < 0.05), while during the DTT, activation was limited to the L-S1 (*p* < 0.05). Intragroup comparisons showed that only the R-FPA exhibited significantly greater activation during the DTT compared to the SST (*p* < 0.05). In the control group, during the SST, activation was observed in the R-S1, bilateral FPA, and R-PSMC (*p* < 0.05). During the CST, activation was observed in the R-IFG and L-PSMC (*p* < 0.05). During the DTT, widespread activation was observed in the R-IFG, L-DLPFC, L-S1, L-FPA, L-PSMC and R-M1 (*p* < 0.05). Intragroup comparisons revealed that activation levels across multiple regions, including bilateral FPA, R-DLPFC, and L-PSMC during the CST, and bilateral S1, bilateral DLPFC, bilateral PSMC, and L-FPA during the DTT, were significantly higher than during the SST (*p* < 0.05). Block-average analysis revealed that the dysphagic group exhibited significantly higher activation in motor and sensory cortical regions during the SST, reduced activation in prefrontal areas during the CST, and limited cortical modulation during the DTT compared to controls (*p* < 0.05).

**Conclusion:**

This fNIRS study revealed distinct cortical activation patterns between post-stroke patients with and without dysphagia. PSD patients demonstrated compensatory hyperactivation in motor-related cortical regions during simple tasks, but exhibited limited modulation capacity under more demanding conditions. In contrast, non-dysphagic patients showed widespread, coordinated cortical activation that scaled with task complexity. These findings suggest that PSD is associated with impaired functional reorganization of cortical swallowing networks.

**Clinical trial registration:**

https://www.chictr.org.cn/, identifier ChiCTR2500098164.

## 1 Introduction

Swallowing is a complex neuromuscular process involving the coordinated activation and inhibition of muscles in the oral cavity, tongue, larynx, pharynx, and esophagus (1). It is regulated by multiple brain regions, including the cerebral cortex, subcortical structures, and brainstem, which together form a complex neural control network (2, 3). This network not only controls basic swallowing movements but also participates in higher-order functions such as executive control, task switching, and cognitive resource allocation, enabling the body to adapt to swallowing demands under different conditions.

Post-stroke dysphagia (PSD) is one of the most common complications of stroke (4). It can lead to aspiration, subsequently causing aspiration pneumonia, and is also a major cause of malnutrition in stroke patients, thereby prolonging hospital stays and increasing readmission rates and mortality risk (5, 6). Patients with dysphagia resulting from supratentorial stroke primarily exhibit impairments in the higher-order control of cortical and subcortical networks (7), including delayed swallowing initiation, coordination disorders (8), and functional deterioration under increased attentional load. Despite significant functional plasticity (9), systematic understanding of the neural compensatory mechanisms and their limitations in PSD patients under different task demands remains limited, hindering the development of precision rehabilitation strategies.

In recent years, various neuroimaging techniques have been used to investigate the neural mechanisms of swallowing, including electroencephalography (EEG), functional magnetic resonance imaging (fMRI), and functional near-infrared spectroscopy (fNIRS). EEG offers excellent temporal resolution at the millisecond level but has limited spatial resolution (10). Although fMRI provides high spatial resolution, it requires subjects to maintain a supine position during scanning, which poses significant aspiration risks for swallowing research (11) and is susceptible to motion artifacts. In contrast, fNIRS, as a non-invasive portable imaging technique, offers several methodological advantages: a favorable balance between spatial and temporal resolution, relative insensitivity to motion artifacts, and portability with cost-effectiveness (12–14). More importantly, fNIRS allows subjects to perform swallowing tasks in a seated or semi-recumbent position, more closely approximating real-world eating conditions and demonstrating good clinical applicability. fNIRS can dynamically monitor hemodynamic changes in swallowing-related cortical regions, providing an important tool for investigating cortical reorganization and rehabilitation potential in stroke patients.

Extensive neuroimaging studies have revealed cortical activation patterns during normal swallowing. A systematic review indicated that the primary motor cortex (M1) and primary somatosensory cortex (S1) are the most consistently activated brain regions during swallowing in healthy adults (11). A recent fNIRS study systematically examined cortical activation during swallowing in healthy individuals and found that nine brain regions, including M1, S1, dorsolateral prefrontal cortex (DLPFC), and premotor and supplementary motor cortex (PSMC), were significantly activated during swallowing. This study demonstrated that M1 and PSMC are involved in regulating the initiation, execution and control of swallowing actions, while DLPFC plays an important role in cognitive activity and attention control during swallowing (15). Additionally, another fNIRS study demonstrated that postural changes during swallowing induce short-term cortical activation changes in sensorimotor regions, further supporting the sensitivity of fNIRS in detecting task-related neural adaptations (16). These findings suggest that cortical control of swallowing involves a complex dynamic network comprising sensory, cognitive, and motor regions, with coordinated activity across these areas ensuring successful swallowing execution. These studies also provide the anatomical basis for fNIRS channel placement in the present study, covering key nodes of the cortical swallowing network, including the prefrontal and sensorimotor cortices.

However, existing research has primarily focused on healthy populations or single-task conditions (17, 18). Moreover, swallowing in daily life is typically continuous and often accompanied by concurrent cognitive tasks, such as conversing or watching television during meals, requiring flexible allocation of cognitive resources (19). Currently, research distinguishing cortical activation patterns between stroke patients with and without dysphagia under varying task complexities remains limited. Therefore, this study employed fNIRS to monitor cortical hemodynamic responses during three swallowing tasks of increasing complexity (single swallowing, continuous swallowing, and video–continuous swallowing dual-task) in stroke patients with and without dysphagia, aiming to preliminarily explore the neural compensatory mechanisms, task adaptability, and functional limitations in PSD patients.

## 2 Materials and methods

### 2.1 Participants

**Figure 1:**
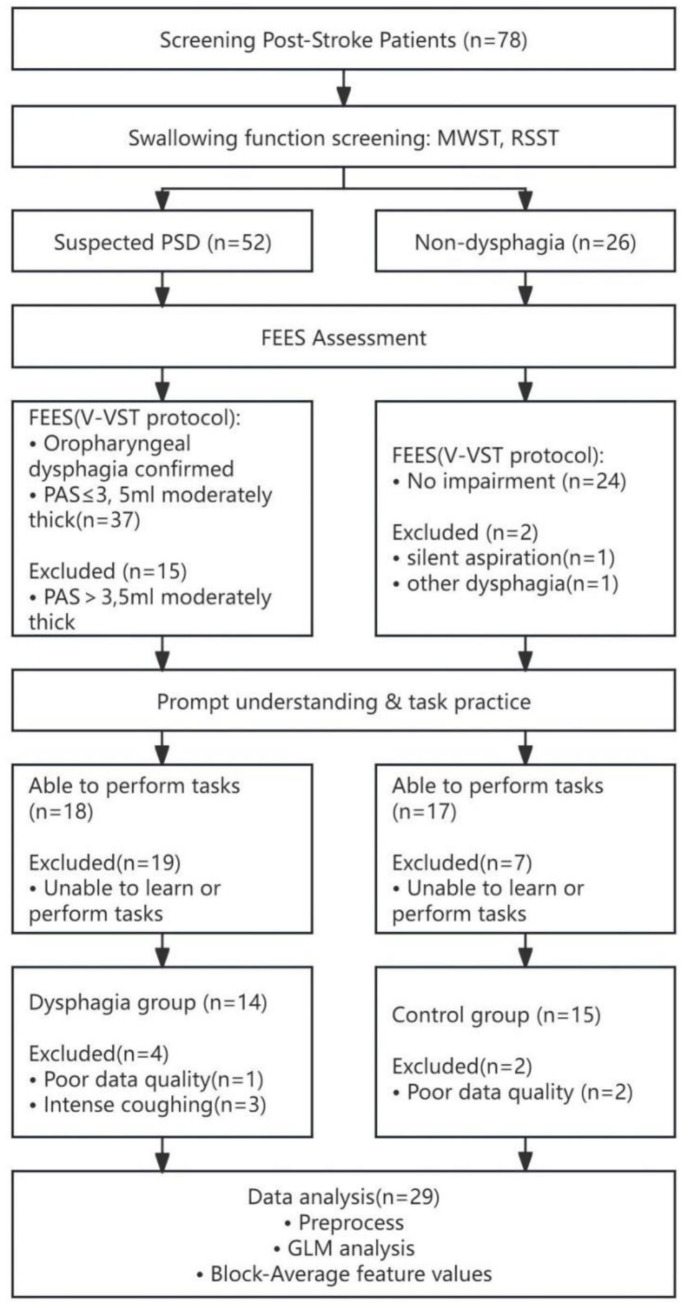
Experimental flow chart.

A total of 29 patients with supratentorial stroke admitted to the Rehabilitation Medicine Department of Binzhou Medical University Hospital from December 2024 to May 2025 were enrolled in this study. These included 14 patients (7 males, 7 females) with dysphagia and 15 patients (9 males, 6 females) without dysphagia (Figure 1). Lesion side (right/left) and lesion location were recorded for all participants. Lesions were categorized as subcortical or cortico-subcortical according to involvement of deep structures and cortical–subcortical regions (Table 1).

**Table 1:** fNIRS channel configuration, brain regions, and function in swallowing.

Channel	S-D pair	MNI coordinates (*X*, *Y*, *Z*)	Brodmann area	Hemisphere (R/L)	Brain region	Function in swallowing
CH1	S1-D1	53, −1, 55	BA6	R	PSMC	Motor planning and preparation
CH2	S1-D6	56, −24, 58	BA2	R	S1	Sensory feedback processing
CH3	S2-D2	55, 43, 2	BA47	R	IFG	Language processing, motor inhibition
CH4	S2-D27	60, 29, 14	BA45	R	PTG	Speech and language processing
CH5	S3-D2	42, 62, 5	BA10	R	FPA	Executive function, attention
CH6	S3-D3	18, 73, 8	BA10	R	FPA	
CH7	S3-D8	30, 65, 20	BA10	R	FPA	
CH8	S4-D3	−12, 73, 10	BA10	L	FPA	
CH9	S4-D4	−35, 63, 11	BA10	L	FPA	
CH10	S4-D9	−21, 66, 25	BA10	L	FPA	
CH11	S5-D4	−52, 42, 10	BA46	L	DLPFC	Cognitive control, working memory
CH12	S5-D10	−55, 28, 24	BA46	L	DLPFC	
CH13	S6-D5	−56, −23, 57	BA2	L	S1	Sensory feedback processing
CH14	S6-D11	−46, −24, 66	BA3	L	S1	
CH15	S7-D1	42, −2, 64	BA6	R	PSMC	Motor planning and preparation
CH16	S7-D6	46, −23, 67	BA3	R	S1	Sensory feedback processing
CH17	S7-D12	34, −24, 74	BA6	R	PSMC	Motor planning and preparation
CH18	S7-D13	31, −4, 69	BA6	R	PSMC	
CH19	S8-D2	48, 50, 19	BA10	R	FPA	Executive function, attention
CH20	S8-D7	52, 36, 30	BA46	R	DLPFC	Cognitive control, working memory
CH21	S8-D8	36, 52, 33	BA9	R	DLPFC	
CH22	S9-D3	8, 69, 25	BA10	R	FPA	Executive function, attention
CH23	S9-D8	16, 61, 35	BA9	R	DLPFC	Cognitive control, decision making
CH24	S9-D9	−8, 61, 38	BA9	L	DLPFC	
CH25	S10-D4	−42, 50, 26	BA10	L	FPA	Executive function, attention
CH26	S10-D9	−28, 52, 37	BA9	L	DLPFC	Cognitive control, decision making
CH27	S10-D10	−46, 35, 37	BA9	L	DLPFC	
CH28	S11-D5	−51, −3, 55	BA6	L	PSMC	Motor planning and preparation
CH29	S11-D11	−40, −1, 63	BA6	L	PSMC	
CH30	S12-D12	21, −25, 77	BA4	R	M1	Primary motor execution
CH31	S12-D13	19, −3, 75	BA6	R	PSMC	Motor planning and preparation
CH32	S13-D11	−35, −22, 73	BA6	L	PSMC	
CH33	S13-D14	−23, −21, 76	BA6	L	PSMC	
CH34	S14-D11	−29, −1, 69	BA6	L	PSMC	
CH35	S14-D14	−18, −2, 74	BA6	L	PSMC	

S-D, source-detector pair; MNI, Montreal Neurological Institute coordinate system; BA, Brodmann area; R/L, right/left hemisphere; PSMC, premotor and supplementary motor cortex; S1, primary somatosensory cortex; M1, primary motor cortex; IFG, inferior frontal gyrus; PTG, pars triangularis of IFG; FPA, frontopolar area; DLPFC, dorsolateral prefrontal cortex.

#### 2.1.1 Inclusion criteria

(1) Patients diagnosed with first-ever supratentorial stroke according to established stroke guidelines, with imaging confirming supratentorial lesion location. (2) Time since stroke onset ≤6 months. (3) Right-handedness confirmed by the Edinburgh Handedness Inventory (EHI). (4) Stable vital signs and a Mini-Mental State Examination (MMSE) score ≥24, indicating patients could understand instructions and cooperate with assessments. (5) No history of dysphagia caused by other neurological diseases, head/neck structural abnormalities, or injuries. (6) No history of neuropsychiatric or psychiatric disorders. (7) Absence of tracheostomy.

#### 2.1.2 Specific criteria for the dysphagia group

Flexible endoscopic evaluation of swallowing (FEES) following a volume-viscosity swallow test (V-VST) protocol (20) confirmed impaired swallowing efficacy and/or safety. Patients were diagnosed with oropharyngeal dysphagia, with a Penetration–Aspiration Scale (PAS) score ≤3 when swallowing 5 mL of moderately thick liquid.

#### 2.1.3 Specific criteria for the control group

FEES following a V-VST protocol confirmed no impairment in swallowing efficacy or safety.

#### 2.1.4 Exclusion criteria

(1) History of previous stroke. (2) Non-stroke-related dysphagia (e.g., dysphagia caused by other neurological or systemic conditions). (3) Auditory or visual impairments. (4) Implanted cardiac pacemakers, intracranial metal implants, cochlear implants, or metallic dentures. (5) Skin lesions on the head or neck, or allergies to electrode pads. (6) Skull defects. (7) History of antidepressant, sedative, or other medications potentially affecting cortical excitability.

#### 2.1.5 Withdrawal criteria

(1) Development of new infarction or hemorrhagic lesions. (2) Inability to tolerate examinations. (3) Withdrawal from the study for other reasons.

All participants provided informed consent and underwent screening according to predefined inclusion and exclusion criteria. The study protocol was approved by the Ethics Committee of Binzhou Medical University Hospital (Approval No. 2024KYLL-092) and registered with the Chinese Clinical Trial Registry (Registration No. ChiCTR2500098164). The registration was completed after participant enrollment but before data analysis.

### 2.2 Experimental preparation and quality control

Before formal data collection, participants briefly practiced all three swallowing tasks with computer-generated prompts to ensure full understanding of the instructions. After the fNIRS headcap was positioned, they rested for 2 min in a comfortable seated position to acclimate to the environment. During recording, standardized instructions and demonstrations were used; participants were asked to remain silent and avoid unnecessary movements, and to maintain natural breathing and a stable head and neck posture during rest. A 5-min break was provided between tasks, with rest periods extended when participants reported marked fatigue. All participants successfully completed all task blocks.

### 2.3 Experimental task paradigm design

**Figure 2:**
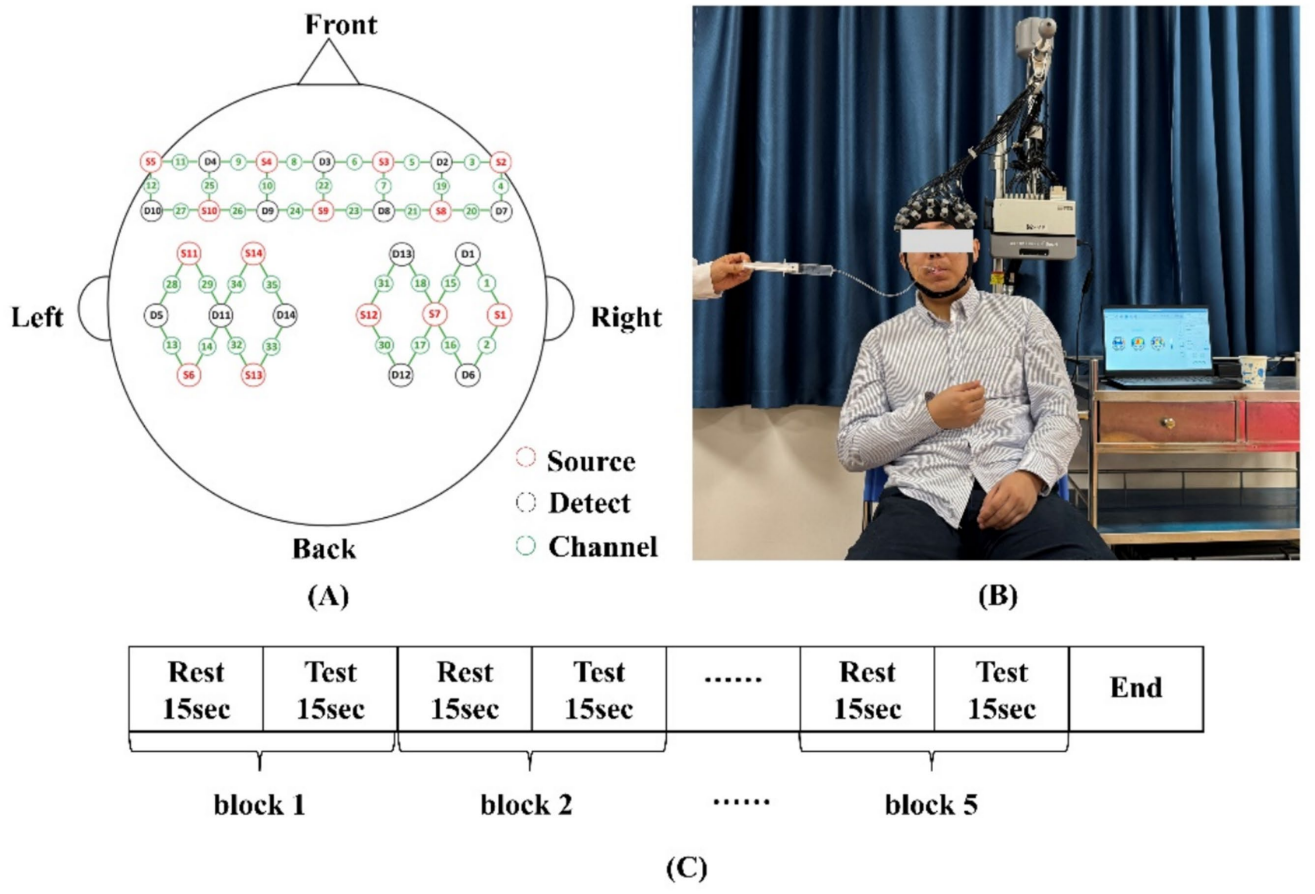
**(A)** Schematic diagram showing the positions of fNIRS channels and probes. **(B)** Experimental set-up. **(C)** Swallowing task paradigm.

A standardized rest-task block design was utilized, with each phase lasting 15 s. This cycle was repeated five times, totaling 150 s. During experimental data collection, the NirSmart system prompted participants with auditory signals indicating the beginning and end of each task. The experimental protocol is illustrated in Figure 2.

The three swallowing tasks were administered in a fixed order from lower to higher cognitive-motor demand (SST → CST → DTT).

Task 1: Single swallowing task (SST).

The experimenter injected 3 mL of moderately thick liquid stimulus into the anterior oral cavity via a syringe. Upon hearing the auditory cue “please swallow,” participants immediately performed a single swallow, ingesting the entire 3 mL bolus at once. Participants relaxed their oral and pharyngeal muscles, maintaining silence when prompted with “please rest.” This procedure was repeated for all five task blocks, resulting in a total liquid intake of 15 mL. Task completion alternated between rest and task phases.

Task 2: Continuous swallowing task (CST).

The experimenter injected 5 mL of moderately thick liquid stimulus into the anterior oral cavity via a syringe. Upon hearing the auditory cue “please swallow continuously,” participants swallowed the liquid in small, successive sips until fully consumed. Participants relaxed their oral and pharyngeal muscles, maintaining silence when prompted with “please rest.” This procedure was repeated for all five task blocks, resulting in a total liquid intake of 25 mL. Task completion alternated between rest and task phases.

Task 3: Video-continuous swallowing dual-task test (DTT).

This task integrated attentional interference with swallowing to simulate cortical activity under complex conditions. A neutral video clip (“General History of China,” duration 2 min 30 s) was used as the visual interference stimulus. The experimenter injected 5 mL of moderately thick liquid stimulus into the anterior oral cavity via a syringe. Upon hearing the auditory cue “please swallow continuously,” participants swallowed the liquid in small, successive sips until fully consumed while simultaneously viewing the video. Participants relaxed their oral and pharyngeal muscles, maintaining silence and stable visual fixation when prompted with “please rest.” This procedure was repeated for all five task blocks, resulting in a total liquid intake of 25 mL. Task completion alternated between rest and task phases, and the video looped continuously during task periods to ensure consistent task duration.

### 2.4 fNIRS measurement

A continuous-wave fNIRS imaging system (NirSmart, Danyang Huichuang Medical Equipment Co., Ltd.) was employed to dynamically monitor cortical activity in participants during three swallowing tasks. The system utilized near-infrared light sources with wavelengths of 730 nm and 850 nm, operating at a sampling frequency of 11 Hz. It comprised 24 emitters and 32 detectors arranged at intervals of 3 cm, forming 35 effective acquisition channels primarily covering the bilateral prefrontal and sensorimotor cortices. The spatial arrangement of fNIRS sources and detectors is illustrated in Figure 2A, and the MNI coordinates with corresponding anatomical regions for each channel are provided in Table 1. This configuration enabled real-time recording of dynamic changes in oxyhemoglobin and deoxyhemoglobin concentrations during swallowing tasks.

### 2.5 fNIRS data analysis

fNIRS data were preprocessed and analyzed using the Homer2 toolbox based on MATLAB.

#### 2.5.1 fNIRS preprocessing

Initially, raw fNIRS intensity data were converted to optical density values. Data points exhibiting signal fluctuations greater than six standard deviations were identified as motion artifacts and corrected using spline interpolation. Subsequently, a bandpass filter (0.01–0.2 Hz) was applied to remove high-frequency physiological noise (e.g., respiration and cardiac activity) and low-frequency baseline drift. Optical density data were then converted into oxyhemoglobin and deoxyhemoglobin concentration changes using the modified Beer–Lambert law. To standardize and ensure comparability across all participants, including those with left- or right-hemisphere lesions, fNIRS functional imaging data from patients with right-hemisphere lesions were horizontally flipped, placing the affected hemisphere uniformly on the left side. Compared to deoxyhemoglobin, oxyhemoglobin concentration changes more sensitively and precisely reflect neuron-induced hemodynamic responses and have been widely employed as critical indicators of local cerebral hemodynamics. Therefore, oxyhemoglobin concentration changes were utilized in this study as the primary measure of cerebral hemodynamic responses.

#### 2.5.2 Cortical activation analysis

A general linear model (GLM) was used to fit and analyze hemodynamic response functions, estimating *β*-values to quantify neural activity levels and patterns across brain regions during specific swallowing tasks. Data from five repeated task blocks under each swallowing condition were analyzed using a block-average method. First, the period from 0–3 s was selected as the baseline, and all task blocks were averaged accordingly. Hemodynamic indices, including central value, mean value, differential value, slope, and integral, were calculated for each channel within task blocks, enabling comprehensive evaluation of cerebral hemodynamic responses during different swallowing tasks.

### 2.6 Statistical analysis

Statistical analyses were performed using SPSS 26.0 software (21). The Shapiro–Wilk test was used to assess normality of distribution. For baseline characteristics, continuous variables with normal distribution were expressed as mean ± standard deviation (SD) and compared using independent-sample *t*-tests; non-normally distributed variables were expressed as median (interquartile range) and compared using the Mann–Whitney *U* test. Categorical variables were presented as frequencies and analyzed using Fisher’s exact test.

For fNIRS data, *β*-values derived from the GLM conformed to a normal distribution and were expressed as mean ± SD. A one-sample *t*-test was conducted to compare *β*-values during each task condition against the baseline value (*β* = 0) to determine significant activation relative to rest. Paired-sample *t*-tests were used for within-group comparisons between different swallowing tasks. Independent-sample *t*-tests were performed to compare activation differences between groups during identical tasks. A *p*-value <0.05 was considered statistically significant.

## 3 Results

### 3.1 Baseline characteristics of participants

A total of 29 stroke patients were included in the final analysis, comprising 14 patients in the dysphagia group and 15 patients in the control group. No significant differences were observed between the two groups in baseline characteristics (age, sex, post-stroke duration, stroke type, lesion side, and lesion location) (*p* > 0.05), indicating comparability between groups (Table 2).

**Table 2:** Demographic and clinical characteristics of participants.

Parameters	Dysphagia group	Control group	Statistic value	*p*
Age (years)	63.50 ± 12.33	56.47 ± 4.87	*t* = 1.994	0.063
Post-stroke duration (days)	25.50 [19.00, 44.25]	36.00 [26.50, 66.00]	*Z* = 1.550	0.121
Sex (male/female)	7/7	9/6		0.715
Stroke type (haemorrhage/infarct)	7/7	9/6		0.715
Lesion side (right/left)	9/5	7/8		0.462
Lesion location (subcortical^a^/cortico-subcortical^b^)	11/3	10/5		0.682

a Subcortical: Lesions restricted to deep structures including basal ganglia, thalamus, corona radiata, internal/external capsule, and centrum semiovale.

b Cortico-subcortical: Lesions involving cerebral cortex with adjacent subcortical structures (e.g., frontal-basal ganglia, multi-lobar lesions), or large (≥20 mm) subcortical lesions.

### 3.2 Brain activation during three swallowing tasks

**Figure 3:**
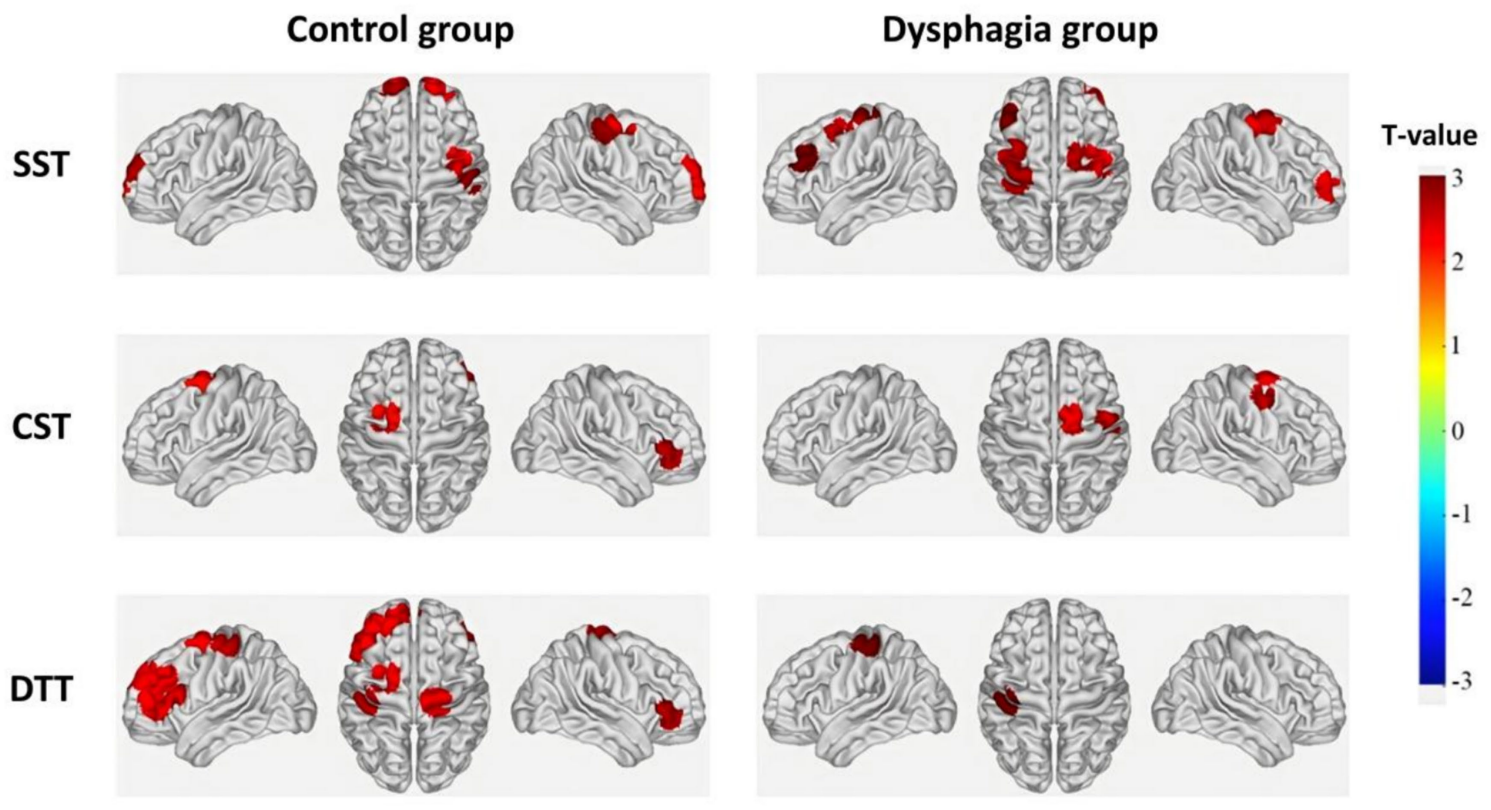
Cortical activation patterns during different swallowing tasks in the dysphagia and control groups. Brain activation maps show channels with significant activation (*p* < 0.05) during the single swallowing task (SST, Task 1), continuous swallowing task (CST, Task 2), and dual-task (DTT, Task 3). The color bar represents *T*-values ranging from −3 to +3, with warmer colors (red) indicating significant positive activation relative to rest.

A GLM and one-sample t-tests were used to analyze cortical activation patterns across the three swallowing tasks. As shown in Figure 3 and Table 3, distinct cortical activation patterns were observed between groups. During the SST, activated channels in the control group included CH2, CH6, CH7, CH8, CH10, and CH15, whereas activated channels in the dysphagia group included CH5, CH15, CH18, CH27, CH29, and CH32. During the CST, activated channels in the control group included CH3 and CH34, while those in the dysphagia group included CH1 and CH31. During the DTT, the control group showed widespread activation involving CH3, CH11, CH12, CH14, CH24, CH25, CH26, CH27, CH30, and CH34; in contrast, the dysphagia group showed activation limited to CH14.

**Table 3:** Significant cortical activation (*β*-values) within dysphagia and control groups during swallowing tasks.

	Control group				Dysphagia group			
Task	Channel	Mean ± SD	*t*	*p*	Channel	Mean ± SD	*t*	*p*
SST	CH2	0.030 ± 0.042	2.805	0.014^*^	CH5	0.033 ± 0.051	2.380	0.033^*^
	CH6	0.025 ± 0.045	2.166	0.048^*^	CH15	0.020 ± 0.032	2.238	0.043^*^
	CH7	0.031 ± 0.054	2.231	0.043^*^	CH18	0.018 ± 0.026	2.586	0.022^*^
	CH8	0.023 ± 0.040	2.247	0.041^*^	CH27	0.024 ± 0.030	2.980	0.011^*^
	CH10	0.026 ± 0.037	2.680	0.018^*^	CH29	0.016 ± 0.024	2.566	0.023^*^
	CH15	0.016 ± 0.029	2.184	0.046^*^	CH32	0.017 ± 0.024	2.631	0.020^*^
CST	CH3	0.044 ± 0.066	2.603	0.021^*^	CH1	0.022 ± 0.032	2.498	0.028^*^
	CH34	0.027 ± 0.049	2.150	0.049^*^	CH31	0.027 ± 0.043	2.284	0.041^*^
DTT	CH3	0.057 ± 0.083	2.650	0.019^*^	CH14	0.014 ± 0.016	4.900	0.016^*^
	CH11	0.030 ± 0.050	2.323	0.036^*^				
	CH12	0.027 ± 0.045	2.393	0.031^*^				
	CH14	0.025 ± 0.038	2.531	0.023^*^				
	CH24	0.020 ± 0.034	2.279	0.039^*^				
	CH25	0.019 ± 0.034	2.200	0.045^*^				
	CH26	0.023 ± 0.039	2.298	0.037^*^				
	CH27	0.036 ± 0.063	2.195	0.045^*^				
	CH30	0.020 ± 0.033	2.361	0.033^*^				
	CH34	0.023 ± 0.041	2.206	0.045^*^				

Only channels with significant activation (*p* < 0.05) are displayed. ^*^p < 0.05.

### 3.3 Comparison of cortical activation patterns under different swallowing tasks

**Figure 4:**
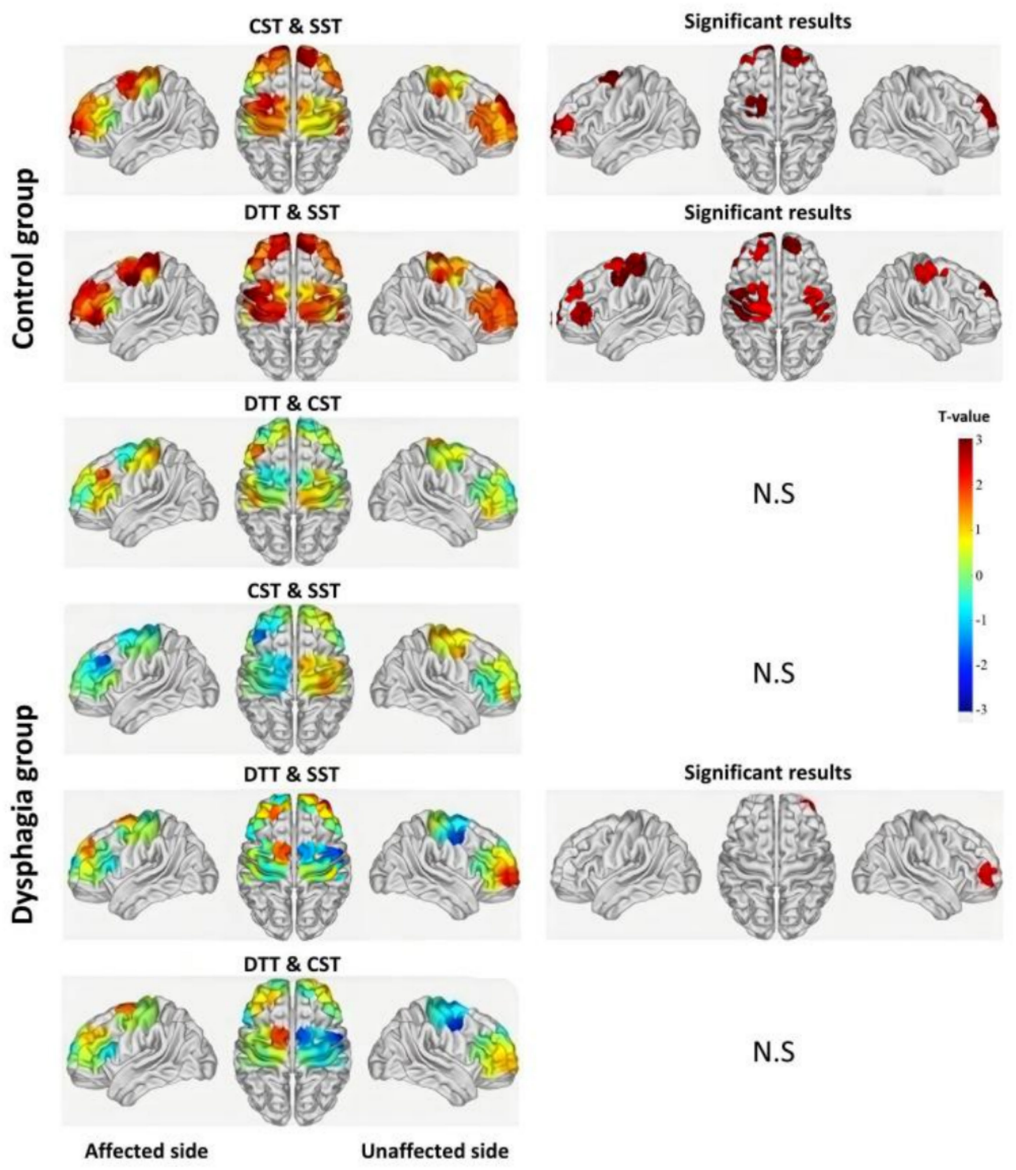
Intragroup comparisons of cortical activation between swallowing tasks. Left panels show *T*-value distributions across the whole brain from paired-sample *t*-tests; right panels show channels with significant differences (*p* < 0.05) or no significant difference (N.S.). Task comparisons include: CST vs. SST, DTT vs. SST, and DTT vs. CST. The color bar represents *T*-values ranging from −3 to +3. N.S., no significant difference.

Intragroup comparisons based on *β*-values were performed to further assess the effects of task difficulty on cortical activation levels. As shown in Figure 4 and Table 4, the control group exhibited significantly increased activation in channels CH7, CH8, CH9, CH22, CH23, and CH34 during the CST compared to the SST (6 channels). During the DTT, activation was more extensive, involving CH2, CH8, CH11, CH14, CH15, CH26, CH28, CH33, and CH34 (9 channels). In contrast, the dysphagia group demonstrated more limited activation changes. Compared with the SST, only CH5 showed a significant increase in activation during the DTT.

**Table 4:** Within-group comparisons of cortical activation (*β*-values) across different swallowing tasks.

Control group						Dysphagia group		
CST&SST			DTT&SST			DTT&SST		
Channel	*t*	*p*	Channel	*t*	*p*	Channel	*t*	*p*
CH7	2.750	0.016^*^	CH2	2.434	0.029^*^	CH5	2.380	0.033^*^
CH8	2.935	0.011^*^	CH8	3.155	0.007^**^			
CH9	2.387	0.032^*^	CH11	2.719	0.017^*^			
CH22	2.425	0.029^*^	CH14	3.247	0.005^**^			
CH23	2.746	0.016^*^	CH15	2.264	0.040^*^			
CH34	2.987	0.010^*^	CH23	2.967	0.010^*^			
			CH26	2.479	0.027^*^			
			CH28	3.363	0.005^**^			
			CH33	2.207	0.045^*^			
			CH34	2.442	0.028^*^			

Only channels with significant differences (*p* < 0.05) are displayed. ^*^p < 0.05 and ^**^p < 0.01.

### 3.4 Intragroup comparison of block-averaged cerebral hemodynamic indices during specific swallowing tasks

**Figure 5:**
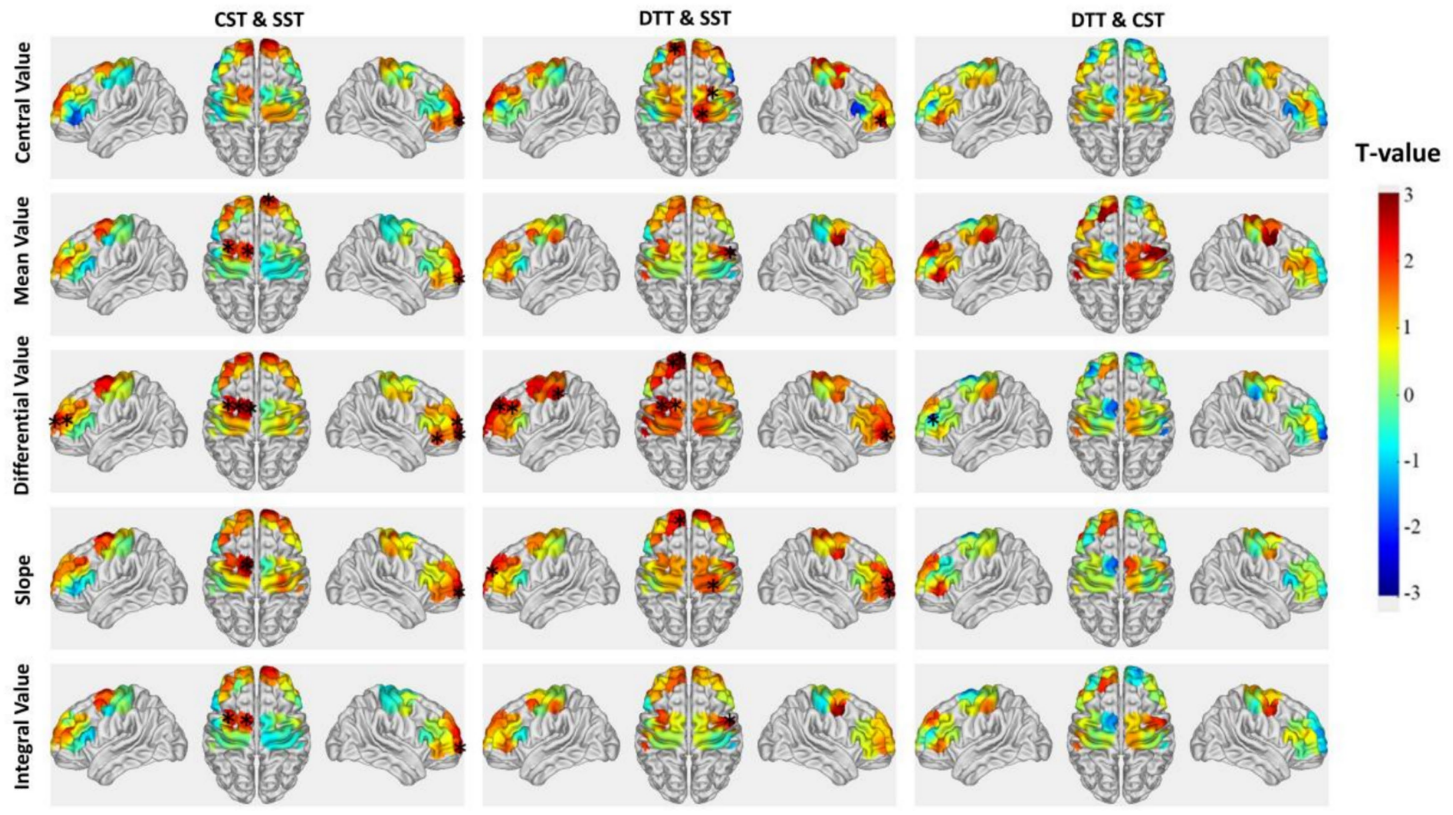
Intragroup comparisons of cerebral hemodynamic indices across swallowing tasks in the control group. Brain maps show *T*-values from paired-sample *t*-tests comparing cerebral hemodynamic indices (central value, mean value, differential value, slope, and integral value) between tasks: CST vs. SST, DTT vs. SST, and DTT vs. CST. The color bar represents *T*-values ranging from −3 to +3, with warm colors (red/yellow) indicating increased values and cool colors (blue) indicating decreased values in the latter task compared to the former. * indicates channels with statistically significant differences (*p* < 0.05).

The intragroup comparison of multiple cerebral hemodynamic indices derived from HbO concentration changes (central value, mean value, differential value, slope, and integral value) across three different tasks, using paired-sample *t*-tests, is presented in Figure 5 and Supplementary Table S1. Significant changes in activation characteristics were observed across tasks in the control group. Compared to the SST, during the CST, there were significant increases in the central values of channels CH6 and CH22, the mean values of CH6, CH22, CH29, and CH35, the differential values of CH3, CH6, CH7, CH10, CH22, CH25, CH29, CH34, and CH35, the slopes of CH6 and CH22, and the integral values of CH6, CH22, CH29, and CH35 (*p* < 0.05). During the DTT, significant increases were observed in the central values of channels CH6, CH10, CH18, and CH30, the mean value of CH1, the differential values of CH5, CH8, CH10, CH13, CH22, CH24, CH26, CH29, and CH34, the slopes of CH6, CH7, CH10, CH17, CH22, and CH24, and the integral value of CH1 (*p* < 0.05). Compared to the CST, the DTT showed a significant decrease in the slope of CH25 (*p* < 0.05).

**Figure 6:**
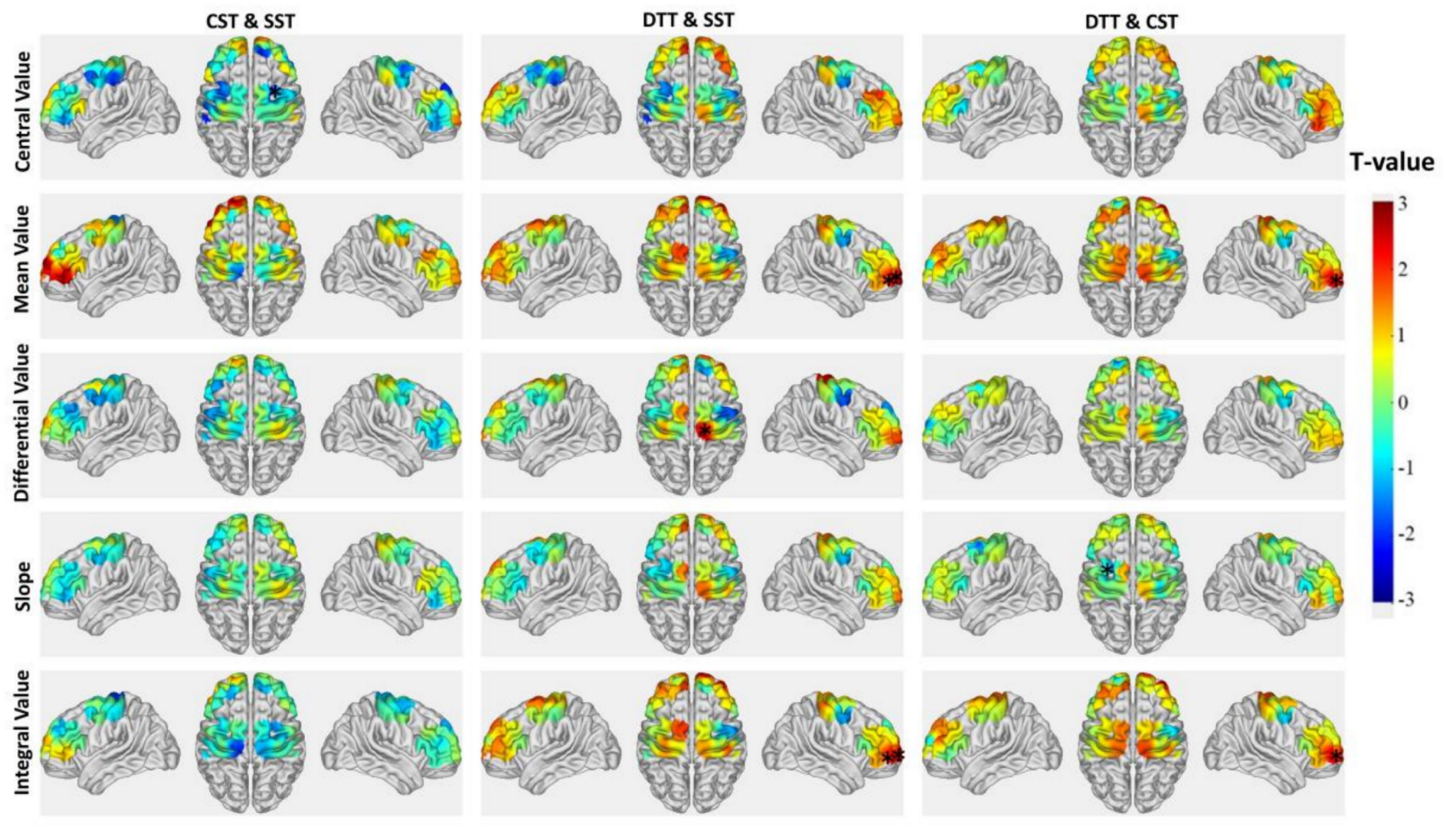
Intragroup comparisons of cerebral hemodynamic indices across swallowing tasks in the dysphagia group. Brain maps show *T*-values from paired-sample *t*-tests comparing cerebral hemodynamic indices (central value, mean value, differential value, slope, and integral value) between tasks: CST vs. SST, DTT vs. SST, and DTT vs. CST. The color bar represents *T*-values ranging from −3 to +3, with warm colors (red/yellow) indicating increased values and cool colors (blue) indicating decreased values in the latter task compared to the former. * indicates channels with statistically significant differences (*p* < 0.05).

In contrast, the dysphagia group showed relatively fewer intragroup changes. Compared to the SST, during the CST, only the central value of channel CH18 significantly decreased (*p* < 0.05). During the DTT, significant increases were observed in the mean and integral values of CH5 and CH6, as well as the differential value of CH30 (*p* < 0.05). Compared to the CST, the DTT showed significant increases in the mean and integral values of CH5 (*p* < 0.05) (Figure 6 and Supplementary Table S1).

### 3.5 Intergroup comparisons of cerebral hemodynamic indices across tasks

**Figure 7:**
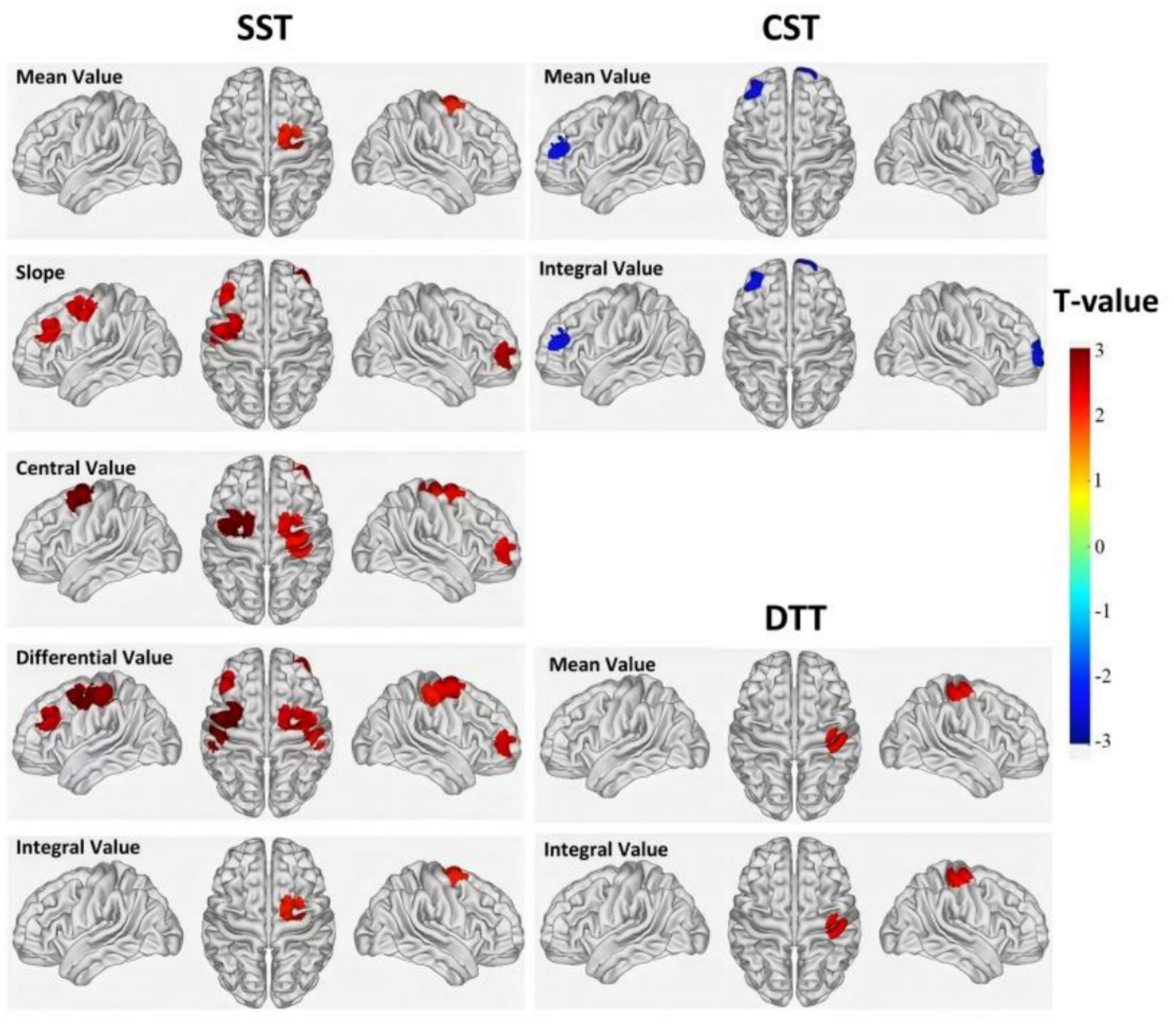
Intergroup comparisons of cerebral hemodynamic indices across swallowing tasks. Brain maps show *T*-values from independent-sample *t*-tests comparing hemodynamic indices between the dysphagia and control groups during the SST, CST, and DTT. The color bar represents *T*-values ranging from −3 to +3, with warm colors (red) indicating greater activation in the control group and cool colors (blue) indicating greater activation in the dysphagia group (*p* < 0.05).

Intergroup comparisons of cerebral hemodynamic indices derived from HbO concentration changes (central value, mean value, differential value, slope, and integral value) between the dysphagia group and the control group across three different tasks, using independent samples t-tests, is presented in Table 5 and Figure 7. During the SST, the dysphagia group exhibited significantly higher central values in channels CH5, CH17, CH18, CH29, and CH34, mean values in channel CH18, differential values in channels CH2, CH5, CH13, CH15, CH18, CH27, CH28, and CH29, slopes in channels CH5, CH27, CH28, and CH29, and integral values in channel CH18 compared to the control group. During the CST, the dysphagia group showed significantly lower mean and integral values in channels CH6 and CH25 compared to the control group. During the DTT, the dysphagia group had significantly higher mean and integral values in channel CH16 compared to the control group.

**Table 5:** Intergroup comparisons of the mean value of cerebral hemodynamic responses across swallowing tasks.

Task	Channel	Brain region	Dysphagia group	Control group	*t*	*p*
SST	CH18	R-PSMC	0.017 ± 0.031	−0.007 ± 0.031	2.074	0.048^*^
CST	CH6	R-FPA	−0.016 ± 0.032	0.013 ± 0.030	−2.456	0.021^*^
	CH25	L-FPA	−0.019 ± 0.040	0.017 ± 0.041	−2.348	0.027^*^
DTT	CH16	R-S1	0.048 ± 0.054	0.003 ± 0.031	2.176	0.044^*^

Data are presented as mean ± SD. ^*^p < 0.05. R-PSMC, right premotor and supplementary motor cortex; R-FPA, right frontopolar area; L-FPA, left frontopolar area; R-S1, right primary somatosensory cortex.

## 4 Discussion

This study revealed distinct cortical activation patterns between PSD patients and stroke patients without dysphagia across swallowing tasks of varying complexity. Using GLM and block-average feature analysis, we identified three key findings. First, during the SST, PSD patients exhibited hyperactivation in multiple cortical regions compared to controls, suggesting compensatory recruitment of neural resources. Second, as task complexity increased, the control group demonstrated progressive expansion of cortical activation (from 6 channels during SST to 10 channels during DTT), whereas the dysphagia group showed a paradoxical reduction (from 6 channels during SST to only 1 channel during DTT). Third, intragroup comparisons revealed that controls exhibited significant task-dependent modulation across multiple channels, while PSD patients showed minimal adaptive changes. These findings suggest that PSD is characterized by initial compensatory hyperactivation but limited capacity to recruit additional neural resources under increased task demands.

Notably, our control group consisted of stroke patients without dysphagia rather than healthy individuals. This design allows us to isolate factors specifically associated with PSD while controlling for the general effects of stroke. Therefore, the observed differences reflect dysphagia-specific neural alterations rather than comparisons with normal physiological swallowing patterns.

### 4.1 Brain activation patterns during swallowing tasks

#### 4.1.1 Differences in brain activation between PSD patients and controls under different swallowing tasks

During the SST, the non-dysphagic stroke control group primarily activated the R-S1, bilateral FPA, and R-PSMC. This activation pattern is broadly consistent with that reported in healthy individuals during water swallowing tasks (16), suggesting relatively preserved sensorimotor integration in non-dysphagic stroke patients.

In contrast, the dysphagia group exhibited notably different activation patterns during the SST, primarily involving bilateral PSMC (right: CH15, CH18; left: CH29, CH32), the R-FPA (CH5), and the L-DLPFC (CH27). The extensive activation of bilateral PSMC suggests that PSD patients require increased recruitment of motor cortical resources to execute even basic swallowing movements (22). The involvement of L-DLPFC warrants particular attention, as this region is not typically activated during simple swallowing tasks in healthy individuals. The DLPFC plays a central role in executive control, working memory, and attentional regulation (23). Its activation during the SST in PSD patients suggests that these patients require greater cognitive control and attentional resources even when performing simple swallowing actions. In healthy individuals, swallowing is often described as a semi-automatic process in which, once initiated, the motor sequence proceeds with minimal conscious oversight (24). The significantly greater L-DLPFC activation in PSD patients compared to controls performing the identical task indicates that PSD patients cannot execute swallowing with the same degree of automaticity, reflecting the initiation of compensatory neural mechanisms (25).

It is important to note that auditory cues were incorporated into the experimental paradigm to ensure temporal synchronization between swallowing tasks and fNIRS data acquisition, consistent with established protocols (17). However, in this study, the auditory cue was only used to signal the onset of each task block rather than to control individual swallowing actions. In the SST, participants initiated swallowing at their own discretion within the task period; in the CST and DTT, participants heard the cue only once at task onset and thereafter swallowed at their own natural pace. This differs from externally paced paradigms that require a cue for each individual swallow, thereby reducing associated cognitive demands. According to Ambrocio et al. (26), self-paced continuous swallowing may represent an intermediate state between fully cued discrete swallowing and spontaneous swallowing, more closely resembling natural drinking behavior. Importantly, since both groups performed identical tasks under the same experimental conditions, the between-group differences in cortical activation cannot be attributed solely to cueing effects, supporting our interpretation that the observed patterns reflect dysphagia-specific neural mechanisms.

Based on the neuroimaging literature, complex dual-task conditions involving swallowing are expected to activate widespread cortical regions, including bilateral DLPFC for executive control, M1 for motor execution, S1 for sensory feedback, and PSMC for motor preparation (15, 27). During the DTT, the non-dysphagic stroke control group demonstrated this expected widespread activation pattern, involving bilateral DLPFC (CH11, CH12, CH24, CH26, CH27), sensorimotor cortices (R-IFG: CH3; L-S1: CH14; R-M1: CH30), and multiple prefrontal and motor preparatory regions (L-FPA: CH25; L-PSMC: CH34). This widespread activation is consistent with previous findings in healthy individuals performing complex swallowing tasks (28). The DLPFC plays a pivotal role as a central hub for executive functions, including task switching, attentional resource allocation, and working memory maintenance (29). The effective L-DLPFC activation observed in our control participants indicates preserved ability to flexibly allocate cognitive resources for concurrent swallowing movements and visual processing during multitasking. Furthermore, M1 activation facilitates precise motor execution (30), while S1 activation provides essential sensory feedback (31).

In contrast, during the DTT, the dysphagia group exhibited activation restricted only to the L-S1 region (CH14). The lack of activation in M1 and PSMC indicates significantly impaired motor initiation and execution capabilities (32). This restricted activation pattern highlights the loss of effective compensation in cognitive-motor dual tasks among PSD patients. The isolated activation of sensory cortical areas suggests that PSD patients concentrate limited neural resources primarily on sensory monitoring due to impaired motor coordination capabilities (33). Thus, PSD patients display constrained neural resource allocation, hindering their ability to simultaneously process multiple tasks as efficiently as controls. The observed shift from hyperactivation during simple tasks to markedly diminished activation during complex tasks demonstrates clear limitations in the compensatory neural mechanisms of PSD patients (11).

#### 4.1.2 Compensatory mechanisms: quantitative evidence from block-average analysis

Compensatory neural mechanisms are critical phenomena observed when comparing PSD patients with non-dysphagic stroke controls during swallowing tasks. While both groups experienced stroke, the between-group differences in cortical activation patterns suggest additional compensatory demands in PSD patients beyond any stroke-related neural reorganization present in the control group. Block-average feature analysis provided additional quantitative evidence regarding compensatory neural load in the dysphagia group by examining activation intensity. During the SST, multiple activation indices, including central, mean, differential, slope, and integral values, of several key channels [e.g., R-FPA (CH5), R-PSMC (CH17, CH18), L-PSMC (CH29)] were significantly higher in the dysphagia group than in the control group (22). These findings indicate that PSD patients require substantially greater neural effort to perform basic swallowing tasks compared to non-dysphagic stroke controls (34). Such hyperactivation reflects compensatory responses to functional deficits, predominantly within the R-FPA and PSMC regions; however, this strategy might represent inefficient compensation, consuming greater neural resources without corresponding improvements in swallowing function (35). This increased cortical activity may originate from cortical dysfunction, disrupted excitation-inhibition balance, and altered synaptic plasticity following stroke (36).

However, a marked shift was observed under more demanding tasks, such as the CST and DTT. It should be noted that all tasks employed the same cueing approach, with auditory cues signaling only task onset and participants swallowing at their own natural pace thereafter. Additionally, 5-min rest periods were provided between tasks to minimize fatigue effects. During the CST, mean and integral activation values for R-FPA (CH6) and L-FPA (CH25) were significantly lower in the dysphagia group compared to controls. This transition from extensive overactivation during simple tasks to selective underactivation during complex tasks suggests a saturation effect in compensatory mechanisms (37), although this interpretation should be considered in the context of the fixed task order (SST → CST → DTT). Despite the rest periods provided, we cannot completely exclude the possibility that accumulated cognitive load from preceding tasks may have contributed to the observed activation patterns. Post-stroke cortical networks possess limited compensatory potential, indicating difficulty in further increasing cortical activation once a ceiling is reached (38). Consequently, the capacity to maintain task performance under increasing complexity is limited. During the DTT, only R-S1 (CH16) showed greater activation in the dysphagia group relative to controls. This selective activation likely reflects a compensatory strategy prioritizing core functions such as sensory monitoring to prevent critical complications (e.g., aspiration), while sacrificing secondary functions, including prefrontal cognitive control, due to impaired motor coordination capacity (34). Nonetheless, this strategy may induce excessive anxiety or fear of swallowing, rather than genuinely enhancing functional recovery (39).

### 4.2 Effects of task difficulty on neuromodulation during swallowing tasks

#### 4.2.1 Control group: hierarchical extension of task-adaptive neuromodulation

The control group exhibited highly adaptive patterns of cortical neuromodulation across swallowing tasks of varying complexity, with significant changes in activation patterns as task difficulty increased. Relative to the SST, brain regions with significantly elevated activation during the CST included bilateral FPA (right: CH7, CH22; left: CH8, CH9), R-DLPFC (CH23), and L-PSMC (CH34). These regions are closely involved in perceptual processing, motor control, and cognitive regulation, reflecting increased demands for motor coordination and rhythmic control during continuous swallowing (40). Activation expanded further during the video-continuous swallowing dual-task, involving additional areas such as sensory cortices (R-S1: CH2; L-S1: CH14), executive function networks (L-DLPFC: CH11, CH26), motor planning and execution regions (R-PSMC: CH15; L-PSMC: CH28, CH33, CH34), and R-M1. These regions collectively underpin sensorimotor processing, motor planning, and execution. Thus, the brain mobilizes extensive neural resources to simultaneously integrate and coordinate swallowing and visual tasks under dual-task conditions.

The hierarchical expansion of activation aligns with the task-demand hypothesis in cognitive neuroscience, which posits that the brain automatically recruits higher-order and more extensive cortical regions as task complexity increases (41, 42). It is particularly noteworthy that activation of the DLPFC not only contributes to executive control but also coordinates complex motor sequences through functional connectivity with the motor cortex (43). In dual-task conditions, the DLPFC coordinates the parallel processing of visual information and swallowing motor execution through its role in the central-executive network (44).

Block-average feature analysis further revealed the fine-tuning of activation intensity within the control group. Compared with the SST, feature values in multiple FPA and PSMC channels significantly increased during continuous swallowing. This enhanced activation expanded further during the video-continuous swallowing dual-task, presenting a coordinated enhancement across the prefrontal-motor cortical network, where differential values of FPA, DLPFC, and PSMC increased synchronously, suggesting the formation of a functional cooperative network (45). Enhanced sensorimotor integration reflects greater demands for precise sensory feedback in dual-task scenarios (46), while specific involvement of M1 highlights its critical role in dual-task execution (15).

The control group demonstrated efficient coordination and execution of dual tasks through widespread cortical activation patterns, particularly within the DLPFC and M1 (25, 47), reflecting flexible allocation of cognitive resources in multitasking contexts. Collectively, these findings depict a dynamic, adaptive pattern in healthy brain networks that flexibly mobilize neural resources in response to task demands to optimize energy efficiency while maintaining functional performance (48).

#### 4.2.2 Dysphagia group: task difficulty and limitations of neuromodulation

In stark contrast to the control group’s hierarchical activation changes, the dysphagia group exhibited markedly limited intragroup activation modulation. No channels showed significantly increased activation during the CST compared with the SST. Likewise, block-average feature comparisons across tasks revealed minimal activation changes, highlighting the restricted compensatory capacity of the dysphagic brain when facing increased task demands.

This severely constrained activation pattern underscores a fundamental deficit in neuromodulation among PSD patients, wherein the brain fails to dynamically adjust activation patterns in response to greater task difficulty due to reduced task adaptability (49), consistent with previous findings demonstrating impaired cortical modulation in stroke patients. The absence of further upregulation under already elevated baseline activation levels likely reflects depleted compensatory reserves (50). Furthermore, impaired neural network plasticity following stroke leads to diminished flexibility and reorganization potential within cortical circuits (25).

In our study, the isolated enhancement observed in R-FPA (CH5) during the dual-task likely represents limited compensatory recruitment, as the right prefrontal cortex attempts to sustain functional output when other cortical regions fail to respond effectively (51). However, such region-specific compensatory activation is insufficient to meet the complex cognitive and motor coordination demands of dual-task conditions, resulting in significant performance deficits in PSD patients (28).

In summary, our findings suggest that PSD patients exhibit impaired task-adaptive neuromodulation, losing the capacity for dynamic cortical regulation in response to changing task demands (52).

### 4.3 Implications for rTMS target selection based on fNIRS findings

The present fNIRS findings may provide preliminary neuroimaging evidence to inform target selection for repetitive transcranial magnetic stimulation (rTMS) in future studies. Our results demonstrated that PSD patients exhibited impaired activation in the DLPFC and M1 during the video-continuous swallowing dual-task, while non-dysphagic controls showed robust activation in these regions. Given that these two areas serve as key nodes in the cortical swallowing network—coordinating executive control and motor output, respectively—they may represent candidate target regions for neuromodulation intervention.

A sequential stimulation approach targeting the DLPFC followed by M1 could be explored in subsequent interventional studies, as this strategy may theoretically address both executive and motor deficits observed in PSD patients (53).

### 4.4 Limitations of this study

This study has several limitations. First, as an exploratory investigation, a formal *a priori* power analysis was not conducted, and the relatively small sample size may affect the representativeness of the results and statistical power. Future confirmatory studies should perform power analyses to determine adequate sample sizes. Second, due to the inherent depth limitation of fNIRS, only superficial cortical activation was measured, and this study did not assess changes in deep brain regions or subcortical structures involved in swallowing control. Third, this study did not include patients with infratentorial stroke; therefore, the findings are primarily applicable to populations with supratentorial lesions. Fourth, the limited sample size precluded subgroup analyses examining the effects of specific lesion characteristics (e.g., lesion volume, precise anatomical location) and disease duration on cortical activation patterns. Future studies with larger cohorts should investigate whether these factors differentially influence cortical reorganization during swallowing. Fifth, our control group consisted of post-stroke patients without dysphagia rather than age-matched healthy individuals. While this design allowed us to control for general stroke-related effects and isolate dysphagia-specific factors, it limits our ability to compare findings with normal swallowing-related cortical activity. Future studies incorporating healthy control groups would provide a more complete understanding of both stroke-related and dysphagia-specific neural changes. Sixth, the three swallowing tasks were administered in a fixed order from lower to higher cognitive–motor demand (SST → CST → DTT), and potential order, practice, and fatigue effects cannot be completely excluded. Future studies with randomized or counterbalanced task sequences are needed to better control for these confounding factors. Seventh, task execution factors may have influenced activation patterns: auditory cues at task onset may have partially contributed to prefrontal activation, and PSD patients typically completed fewer swallows during continuous tasks due to impaired coordination. Future studies employing event-related designs or controlling for swallowing frequency could further isolate swallowing-specific cortical activation. Eighth, swallowing events were monitored solely through visual observation of laryngeal movement without concurrent surface electromyography (sEMG) or video recording to objectively verify swallowing timing and frequency. Future studies should consider incorporating sEMG and/or respiratory monitoring to provide objective verification of swallowing events and to enable more precise temporal alignment between swallowing actions and hemodynamic responses. Ninth, multiple comparison corrections such as FDR or Bonferroni were not applied in the multi-channel comparisons, which may increase the risk of false positives; future studies should employ appropriate correction methods to enhance the robustness of statistical conclusions. Tenth, as a single-center cross-sectional study, the generalizability of findings may be limited, and we were unable to track dynamic changes in cortical activation patterns during the recovery process. Finally, this study did not investigate the correlation between cortical activation and clinical swallowing function. Future research should expand the sample size and incorporate multimodal imaging, longitudinal designs, and functional assessments to more comprehensively elucidate the neural mechanisms underlying post-stroke dysphagia and optimize rehabilitation strategies.

## 5 Conclusion

In conclusion, this fNIRS study revealed distinct cortical activation patterns between post-stroke patients with and without dysphagia during swallowing tasks of varying complexity. PSD patients demonstrated compensatory hyperactivation in motor-related cortical regions during simple tasks, but exhibited limited modulation capacity and signs of decompensation under more demanding conditions. In contrast, non-dysphagic patients showed widespread, coordinated cortical activation that scaled with task complexity. These findings suggest that PSD is associated with impaired functional reorganization of cortical swallowing networks. The observed activation patterns in the DLPFC and sensorimotor regions may provide preliminary neuroimaging evidence for future studies exploring targeted neuromodulation approaches. However, further research, including randomized controlled trials, is needed to validate these findings and determine the clinical efficacy of any specific intervention strategy.

## Data Availability

The raw data supporting the conclusions of this article will be made available by the authors, without undue reservation.
