# Supplementary material for: Cortical activation characteristics during different swallowing tasks in post-stroke patients: a functional near-infrared spectroscopy study
Source: Front Neurol. 2026 Jan 15;16:1733949. doi: 10.3389/fneur.2025.1733949 (PMC12852004; doi:10.3389/fneur.2025.1733949)
Supplement: Supplementary file 1 [file Table_1.docx]

**Table S1 Intragroup comparison of block-averaged cerebral hemodynamic indices during specific swallowing tasks**

|  | **Channel** | **Central Value** | | **Mean Value** | | **Differential Value** | | **Slope** | | **Integral Value** | |
| --- | --- | --- | --- | --- | --- | --- | --- | --- | --- | --- | --- |
|  |  | ***t*** | ***p*** | ***t*** | ***p*** | ***t*** | ***p*** | ***t*** | ***p*** | ***t*** | ***p*** |
| **Control group** |  |  |  |  |  |  |  |  |  |  |  |
| **CST vs SST** | ch3 | 1.649 | 0.121 | 1.318 | 0.209 | 2.268 | 0.040* | 1.537 | 0.147 | 1.311 | 0.211 |
|  | ch5 | 1.361 | 0.195 | 0.858 | 0.405 | 1.766 | 0.099 | 1.737 | 0.104 | 0.855 | 0.407 |
|  | ch6 | 2.894 | 0.011* | 2.312 | 0.037* | 3.424 | 0.004* | 2.965 | 0.010* | 2.306 | 0.037* |
|  | ch7 | 2.086 | 0.056 | 2.139 | 0.051 | 2.215 | 0.044* | 1.698 | 0.112 | 2.138 | 0.051 |
|  | ch10 | 2.090 | 0.055 | 1.731 | 0.106 | 3.079 | 0.008* | 1.693 | 0.113 | 1.728 | 0.106 |
|  | ch22 | 2.799 | 0.014* | 2.373 | 0.033* | 2.804 | 0.014* | 2.395 | 0.031* | 2.370 | 0.033* |
|  | ch25 | -0.801 | 0.437 | 2.058 | 0.059 | 2.532 | 0.024* | 1.209 | 0.247 | 2.060 | 0.058 |
|  | ch29 | 0.612 | 0.551 | 2.327 | 0.035* | 2.700 | 0.017* | 1.947 | 0.072 | 2.325 | 0.036* |
|  | ch34 | 1.590 | 0.134 | 1.467 | 0.164 | 2.632 | 0.020* | 1.382 | 0.189 | 1.467 | 0.164 |
|  | ch35 | 1.638 | 0.124 | 2.308 | 0.037* | 3.504 | 0.004* | 3.837 | 0.002* | 2.306 | 0.037* |
| **DTT vs CST** | ch1 | 1.943 | 0.072 | 2.812 | 0.014* | 1.704 | 0.110 | 2.098 | 0.055 | 2.805 | 0.014* |
|  | ch5 | 2.622 | 0.020* | 1.600 | 0.132 | 2.751 | 0.016* | 1.952 | 0.071 | 1.594 | 0.133 |
|  | ch6 | 0.553 | 0.589 | 0.944 | 0.361 | 1.616 | 0.128 | 2.655 | 0.019* | 0.935 | 0.366 |
|  | ch7 | 0.882 | 0.393 | 0.972 | 0.348 | 1.747 | 0.103 | 2.335 | 0.035* | 0.965 | 0.351 |
|  | ch8 | 0.028 | 0.978 | 1.167 | 0.263 | 2.635 | 0.020* | 2.144 | 0.050 | 1.162 | 0.265 |
|  | ch10 | 3.204 | 0.006* | 1.395 | 0.185 | 2.510 | 0.025* | 2.255 | 0.041* | 1.388 | 0.187 |
|  | ch13 | -0.102 | 0.920 | 1.923 | 0.075 | 2.603 | 0.021* | 0.547 | 0.593 | 1.922 | 0.075 |
|  | ch17 | 0.812 | 0.431 | -0.371 | 0.716 | 1.587 | 0.135 | 2.153 | 0.049* | -0.377 | 0.712 |
|  | ch18 | 2.727 | 0.016* | 1.369 | 0.193 | 1.609 | 0.130 | 1.459 | 0.167 | 1.367 | 0.193 |
|  | ch22 | 1.930 | 0.074 | 1.980 | 0.068 | 2.913 | 0.011* | 2.855 | 0.013* | 1.976 | 0.068 |
|  | ch23 | 1.594 | 0.133 | 1.045 | 0.314 | 1.049 | 0.312 | 0.486 | 0.635 | 1.043 | 0.315 |
|  | ch24 | 2.081 | 0.056 | 1.794 | 0.094 | 3.161 | 0.007* | 2.189 | 0.046* | 1.790 | 0.095 |
|  | ch26 | 1.757 | 0.101 | 1.658 | 0.119 | 2.371 | 0.033* | 2.118 | 0.053 | 1.653 | 0.121 |
|  | ch29 | 1.174 | 0.260 | 2.074 | 0.057 | 2.422 | 0.030* | 1.529 | 0.148 | 2.070 | 0.057 |
|  | ch30 | 2.320 | 0.036* | 0.501 | 0.624 | 1.904 | 0.078 | 1.690 | 0.113 | 0.494 | 0.629 |
|  | ch34 | 2.005 | 0.065 | 1.012 | 0.329 | 2.686 | 0.018* | 1.610 | 0.130 | 1.007 | 0.331 |
| **DTT vs CST** | ch25 | -1.751 | 0.102 | -1.071 | 0.302 | -2.159 | 0.049* | -0.401 | 0.694 | -1.078 | 0.299 |
| **Dysphagia group** | | | | |  |  |  |  |  |  |  |
| **CST vs SST** | ch18 | -2.105 | 0.045* | -1.424 | 0.167 | -1.360 | 0.186 | -0.316 | 0.754 | -1.422 | 0.167 |
| **DTT vs SST** | ch5 | 0.833 | 0.417 | 2.183 | 0.044* | 1.580 | 0.134 | 0.160 | 0.875 | 2.183 | 0.044* |
|  | ch6 | 1.681 | 0.112 | 2.610 | 0.019* | 1.896 | 0.076 | 1.569 | 0.136 | 2.609 | 0.019* |
|  | ch30 | 1.336 | 0.200 | 1.295 | 0.214 | 2.386 | 0.030* | 1.651 | 0.118 | 1.294 | 0.214 |
| **DTT vs CST** | ch5 | 1.011 | 0.328 | 2.278 | 0.038* | 1.061 | 0.305 | -0.283 | 0.781 | 2.277 | 0.038* |
|  | ch34 | -1.320 | 0.207 | 0.937 | 0.364 | -1.148 | 0.269 | -2.274 | 0.038* | 0.939 | 0.363 |

**Note:** The intragroup comparison of multiple cerebral hemodynamic indices derived from HbO concentration changes (central value, mean value, differential value, slope, and integral value). Paired t-tests were used to compare HbO responses between different tasks within each group. Only channels with at least one significant difference (*p* < 0.05) are displayed. **p* < 0.05.
